# Fusion versus fixation in complex comminuted C3-type tibial pilon fractures: a systematic review

**DOI:** 10.1186/s13037-021-00298-2

**Published:** 2021-10-18

**Authors:** Yash P. Chaudhry, Efstratios Papadelis, Hunter Hayes, Philip F. Stahel, Erik A. Hasenboehler

**Affiliations:** 1 Department of Orthopaedic Surgery, Philadelphia College of Osteopathic Medicine, Philadelphia, PA 19131 USA; 2 Department of Specialty Medicine, Rocky Vista University, College of Osteopathic Medicine, Parker, CO 80134 USA; 3 Department of Orthopaedic Surgery, The Johns Hopkins University, Johns Hopkins Bayview Medical Center, 4940 Eastern Ave. #A667, Baltimore, MD 21224 USA

**Keywords:** Tibial pilon fracture, Primary ankle arthrodesis, Staged surgical fixation, Patient outcomes, Post-traumatic osteoarthritis, Complications

## Abstract

**Background:**

Comminuted intra-articular tibial pilon fractures can be challenging to manage, with high revision rates and poor functional outcomes. This study reviewed [1] treatment, complications, and clinical outcomes in studies of complex comminuted tibial pilon fractures (type AO43-C3); and [2] primary ankle arthrodesis as a management option for these types of complex injuries.

**Methods:**

A systematic literature search was performed on PubMed from 1990 to 2020 to determine complications and outcomes after staged fracture fixation and primary ankle joint arthrodesis for comminuted C3-type tibial pilon fractures.

The search was conducted in compliance with the PRISMA guidelines, using the following MeSH terms: “tibial pilon”/“pilon fracture”/“plafond fracture”/“distal tibial”/“43-C3”/“ankle fracture”/“ankle fusion”/“primary ankle arthrodesis”/“pilon fracture staged”/“pilon external fixation” and “pilon open reduction internal fixation.” Inclusion criteria were restricted to original articles in English language on adult patients ≥18 years of age. Eligibility criteria for retrieved publications were determined using a “PICO” approach (population, intervention/exposure, comparison, outcomes). Weighted analysis was used to compare treatment groups on time to definitive treatment, follow-up time, range of motion, fracture classification, and complications.

**Results:**

The systematic literature review using the defined MeSH terms yielded 72 original articles. Of these, 13 articles met the eligibility criteria based on the PICO statements, of which 8 publications investigated the outcomes of a staged fixation approach in 308 cumulative patients, and 5 articles focused on primary ankle arthrodesis in 69 cumulative patients. For staged treatment, the mean wound complication rate was 14.6%, and the malunion/nonunion rate was 9.9%. For primary arthrodesis, the mean wound complication rate was 2.9%, and the malunion/nonunion rate was 2.9%. After risk stratification for fracture type and severity, the small cumulative cohort of patients included in the primary arthrodesis publications did not provide sufficient power to determine a clinically relevant difference in complications and long-term patient outcomes compared to the staged surgical fixation group.

**Conclusions:**

At present, there is insufficient evidence in the published literature to provide guidance towards consideration of ankle arthrodesis for complex comminuted C3-type tibial pilon fractures, compared to the standard treatment by staged surgical fracture fixation.

## Background

Tibial pilon fractures are complex injuries that typically result from high-energy trauma mechanisms, e.g. axial compression or shearing injuries from motor vehicle accidents or falls from heights [1]. Accounting for less than 10% of all tibial fractures [2, 3], they can be challenging to manage because of the reconstruction needed of the involved articular surface and adjacent metaphysis and fibula fracture which is observed in up to 85% of cases.

Several classification systems allow comparison among different series and help guide clinical decision making. The AO/OTA Classification system [4, 5] is the most comprehensive system for categorizing pilon fractures, organizing them according to the degrees of articular surface involvement and comminution [6, 7]. Type AO 43-C3 fractures, which have a high degree of comminution and extensive intra-articular involvement, are the most challenging because of the severe disruption to the articular surface and the associated soft tissue injury [3, 8–11].

Studies [1–3, 8, 12] have shown that complications and outcomes are closely associated with the degree of injury, especially that of soft tissues. Therefore, the goal of treatment is not only to stabilize the fracture and reduce the joint anatomically, but to preserve the soft tissue envelope [1–3, 8]. This concept has led to recommendations of a staged procedure especially for high-energy AO 43-C2 and AO 43-C3 injuries; initial external fixation (with or without internal fixation of the fibula) to allow soft tissue resting followed by open reduction and internal fixation (ORIF) 10 to 14 days later [10–15].

Regardless of the fixation method used to treat pilon fractures, a substantial percentage of patients develop complications postoperatively, including wound infection, osteomyelitis, nonunion, and post-traumatic osteoarthritis (PTOA) [10]. Although operative techniques and complication rates in pilon fractures have improved, the reported complication rates may lead to the erroneous conclusion that the outcomes for these injuries are substantially better than what is experienced clinically. These complications have the potential to result in long hospital stays, need for additional surgeries (e.g., secondary arthrodesis), prolonged pain control, non-weightbearing periods and absence from work.

The hypothesis of the present study was to review the literature of the past 30 years to assess treatment modalities, complications, and clinical outcomes for AO 43-C3 tibial fractures treated with a staged approach, versus primary arthrodesis to compare the outcomes between these 2 patient populations and to determine if primary arthrodesis can be a definitive treatment option for these fractures.

## Methods

### Literature review process

This study was conducted in accordance with the Preferred Reporting Items for Systematic Reviews and Meta-Analyses (PRISMA) guidelines [16]. The authors searched the PubMed database for articles from January 1990 through June 2020 containing any of the following MeSH terms or combinations thereof: “tibial pilon,” “pilon fracture,” “plafond fracture,” “distal tibial,” “43-C3,” “ankle fracture,” “ankle fusion,” “primary ankle arthrodesis,” “pilon fracture staged,” “pilon external fixation,” and “pilon open reduction internal fixation.” Studies were considered eligible and included in the review if they met the following criteria, designed through the population, intervention/exposure, comparison, and outcome (PICO) approach [17]:

1. *Population*: adult patients who underwent surgery for AO 43-C3 tibial pilon fractures
2. *Intervention/Exposure*: randomized controlled trials (RCTs), prospective cohort studies, and retrospective cohort studies examining outcomes of operative fixation of AO 43-C3 tibial pilon fractures with either staged treatment or primary arthrodesis
3. *Comparison*: outcomes for patients receiving staged treatment versus primary arthrodesis
4. *Outcomes*: outcome measures included rates of malunion, nonunion, wound infection, pain from implant, development of PTOA, amputation, secondary ankle arthrodesis, as well as ankle range of motion

#### PICO question 1

In adult patients with AO 43-C3 type tibial pilon fractures, should primary arthrodesis be performed (versus staged treatment) to decrease rates of short-term complications in the form of malunion, nonunion, wound infection, and hardware-related pain?

#### PICO question 2

In adult patients with AO 43-C3 type tibial pilon fractures, should primary arthrodesis be performed (versus staged treatment) to decrease rates of long-term complications in the form of development of PTOA, requirement for amputation or secondary ankle arthrodesis, and improved ankle range of motion?

Other exclusion criteria included animal and in vitro studies, articles published in languages other than English, case reports and case series of fewer than 5 patients, studies that used the Ruedi-Allgower [18] rather than the AO classification system, articles that pooled their data without clearly distinguishing among specific outcomes of different fracture patterns, and articles missing clear outcome parameters or follow-up information.

Abstracts and full-text manuscripts were screened and reviewed by three authors independently (YPC, HH, EP). Any discrepancies found were resolved by cross review and discussion among the authors until consensus was reached.

### Risk of Bias assessment

The risk of bias was assessed within each study using the methodological index for non-randomized studies (MINORS) criteria [19]. The MINORS criteria is graded on a scale of 16 for noncomparative studies and 24 for comparative studies, and has demonstrated good test-retest reliability and internal consistency in the examination of non-randomized studies in meta-analyses and systematic reviews [19].

### Statistical analysis

The authors classified treatment groups in the included studies according to the use of fusion procedures. Five studies [8, 20–23] used fusion procedures, 8 studies used other types of treatment [3, 15, 24–29] (Table 1). Weighted analysis, using sample sizes reported in each article, was used to compare these 2 patient groups on key indices. These indices were time to definitive treatment, follow-up time, range of motion, fracture classification (fracture type and open vs closed), and presence of complications.

**Table 1:** Studies of AO-43C Tibial Pilon Fractures Treated with Primary Arthrodesis or Staged Treatment Using the AO/Orthopedic Trauma Association Classification System

Study	Treatment	Mean Time to Definitive Treatment (d)	Fxs(n)	AO/OTA 43 Fx Type (N)		Open Fxs (n)	Complications			Amp (n)	Mean ROM (°)	Secondary Fusion (n)	Mean Follow-up (mo)
				C1/C2	C3		MU/NU (n)	W/I/P (n)	PTOA(n)^a^				
Beaman and Gellman [20]	Temporary ex fix followed by primary arthrodesis	15	12	0	12	5	0	1	NR	0	NR	0	24
Borens et al. [3]	2-Staged, minimally invasive anterior approach	11.3	17	9	8	3	0	6	8	0	30	1	17
Bozic et al. [8]	Temporary ex fix followed by primary arthrodesis	140	15	0	15	8	0	1	NR	0	NR	0	39
Grose et al. [28]	2-Staged, lateral approach	NR	43	8	27	18	4	9	NR	0	43	0	13.7
McCann et al. [29]	Primary ORIF or 2-staged	13.6	48	29	6	3	1	9	5	NR	NR	NR	9.1
Morgan et al. [21]	Temporary ex fix followed by late primary arthrodesis	280	6	0	5	4	0	0	NR	0	NR	0	35
Sirkin et al.(15)^b^	2-Staged, ex fix followed by ORIF (closed)	12.7	22	14	42	22	NR	6	NR	0	NR	0	NR
	2-Staged, ex fix followed by ORIF (open)	14	34			34	NR	4	NR	1	NR	1	NR
Zelle et al. [22]	Temporary ex fix followed by primary arthrodesis	22	20	4	16	5	1	1	NR	0	NR	0	86
Guan et al. [25]	2-staged	19	13	0	13	3	2	0	NR	NR	28	NR	32
Chen et al. [24]	3-staged (ex fix, ORIF posterior column, ORIF anterior/medial columns)	12	25	0	25	3	0	2	2	NR	NR	0	24.4
Wang et al. [26]	Temporary ex fix followed by ORIF (vacuum sealing drainage)	14.3	16	6	10	NR	0	0	0	0	R	NR	23.3
Leonetti et al. [27]	Ex fix vs. ORIF	NR	71	9	14	20	13	9	14	NR	NR	NR	36
Beckwitt et al. [23]	Primary ORIF and primary arthrodesis	NR	35	0	35	9	6	1	11	NR	NR	1	73.7
Total or weighted mean		16.1	377	79	228	103	27	49	40	3	37.3	3	33.2

*Amp, amputation; Fxs, fractures; ex fix, external fixation; MU/NU, malunion, nonunion; NR, not reported; ORIF, open reduction and internal fixation; ROM, range of motion; W/I/P, wound problem, infection, or pain from implant*

^a^ Symptomatic or radiographic

^b^ Two fracture groups reported separately—closed and open—with 56 fractures in 53 patients

## Results

**Fig. 1:**
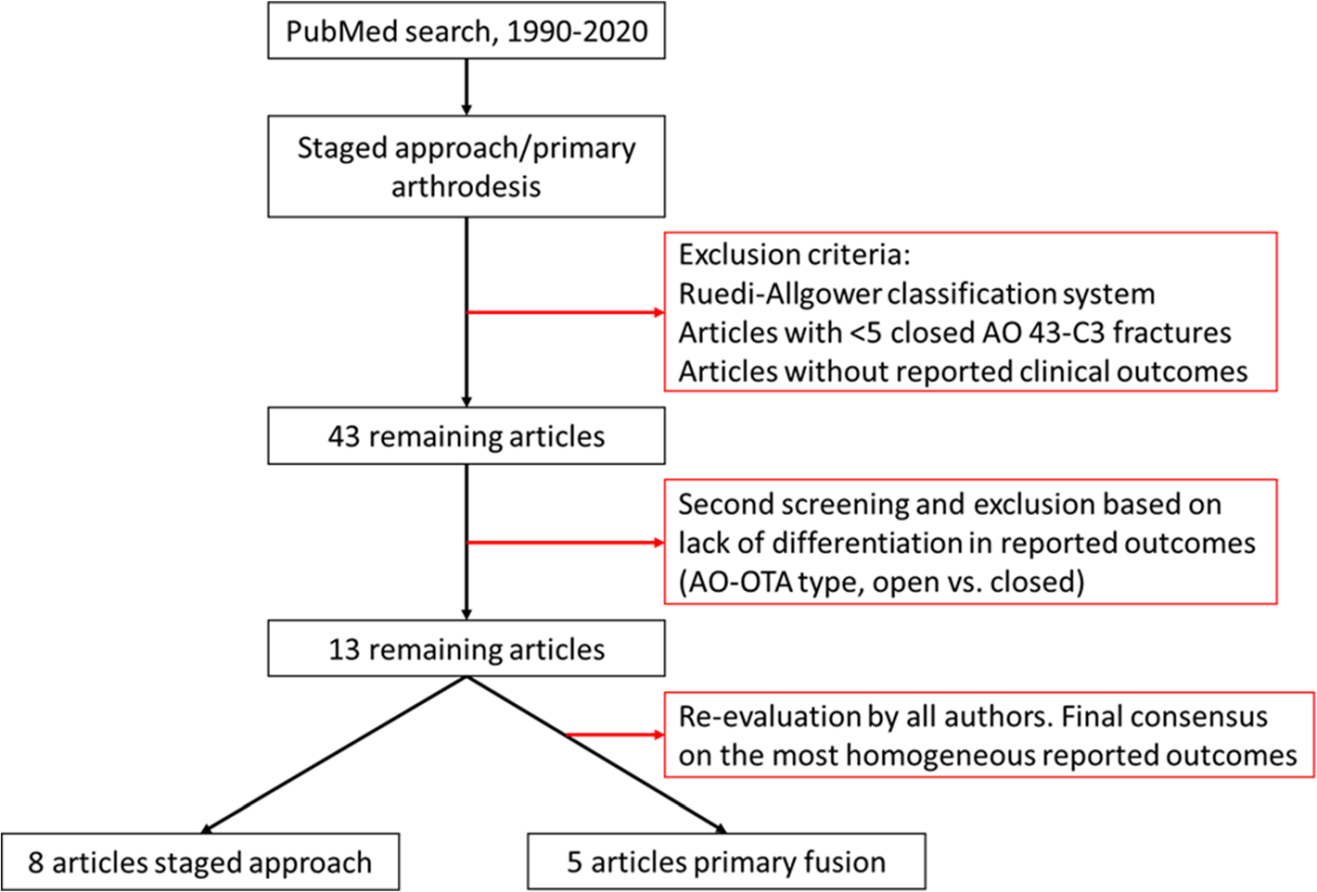
Flow chart of study selection

The PICO questions addressed in this study were to evaluate the effectiveness of primary arthrodesis as a treatment option in AO 43-C3 type tibial pilon fractures as an alternative to staged treatment in reducing the rates of postoperative short-term complications and poor long-term outcomes. The initial MeSH keyword search yielded 72 original articles, 29 of which were eliminated based on the exclusion criteria (Fig. 1). The full-length manuscripts of the remaining 43 articles were included for review. None of the 43 articles had complete information in all the PICO categories investigated (Table 1). For articles reporting a staged surgical fixation approach, the most common reason for exclusion from the final review was the lack of differentiation in reported outcomes with regard to AO-OTA fracture type and status of the skin (open vs closed fractures), which accounted for the exclusion of 30 articles. In these articles, the outcomes were reported as the total number of the complication of interest for 1 treatment group compared with another treatment group, but without distinction as to whether the complication occurred in AO 43-C1 or AO 43-C3 injury or the status of the skin at the time of injury. The remaining 13, with 8 articles reporting a staged approach [3, 15, 24–29] and 5 articles pertaining to primary arthrodesis [8, 20–23] were evaluated and analyzed. The 13 studies examined in this review included 377 total fractures, 228 of which involved AO 43-C3 pilon fractures.

Compared with cases treated without fusion, those treated with fusion had a significantly lower proportion of C1/C2 fractures (*P* < .001), but a higher proportion of C3 fractures (92.8% vs. 53.2%) (*P* < .001). The fusion cohort also had a significantly higher period of follow-up (*P* = .013) (Table 2). There were no statistically significant differences between these two cohorts with regards to time to definitive treatment (*P* = .063) or proportion of open fractures (*P* = .594). None of the 13 studies examined all the assessed variables in this review. Only one of the studies was a comparative study [23], the rest were case series.

**Table 2:** Comparison of primary arthrodesis and staged treatment for AO 43-C Tibial Pilon Fractures

	Primary Arthrodesis (*n* = 69 patients)	Staged Treatment (*n* = 308 patients)	*P* Value
Number of publications	5	8	N/A
Time to definitive treatment, days^a^	82.02 (44.48)	13.58 (0.68)	.063
Follow-up time, months^a^	59.82 (11.85)	25.89 (5.45)	.013
Range of motion, degrees^a^	NA	37.30 (2.44)	N/A
Open fracture^b^	39.1	37.8	.594
Complications			
Malunion/non-union^b^	2.9	9.9	.085
Wound problem/infection/pain^b^	5.7	14.6	.049
PTOA^b^	NA	20.4	1.00
Amputation^b^	0	0.08	1.00

N/A, not applicable

^a^Expressed as mean (±SEM)

^b^Expressed as percentage

### PICO question 1: short-term complications

Twenty-seven cases from the reviewed studies involved the development of malunion/nonunion and 49 involved wound problems/infection or pain from implant. The fusion cohort had a significantly lower proportion of cases involving wound issues or symptomatic hardware (*P* = .049), but no significant differences were observed in the rates of malunion/nonunion between the two cohorts (*P* = .085).

### PICO question 2: long-term complications

Forty cases were noted to have developed PTOA; however all of these were observed in the staged treatment cohort as this was not recorded for any of the arthrodesis cases reviewed. Three cases involved the need for subsequent amputation of the affected extremity during the follow-up period; all three were in the staged treatment cohort as well (0.08% incidence).

### Risk of Bias assessment

Of the noncomparative studies, the mean MINORS score was 10.2 (standard deviation 1.7). The single comparative study by Beckwitt et al. had a MINORS score of 19. Results of the MINORS scoring is summarized on Table 3.

**Table 3:** Quality assessment of non-randomized studies using the Methodological Index for Non-Randomized Studies (MINORS)

Study	Total MINORS Score	Clearly Stated Aim	Consecutive Patients	Prospective Data Collection	Appropriate Endpoint	Unbiased Evaluation of Endpoint	Appropriate Follow Up	Loss to Follow Up	Prospective Calculation of Sample Size	Gold Standard Control	Contemporary Groups	Baseline Equivalence of Groups	Statistical Analyses
Beaman and Gellman	12	2	2	1	2	1	2	2	0	–	–	–	–
Borens et al	11	1	2	1	2	1	2	2	0	–	–	–	–
Bozic et al	8	1	0	1	1	1	2	2	0	–	–	–	–
Grose et al	12	2	2	1	2	1	2	2	0	–	–	–	–
McCann et al	10	1	2	1	1	1	2	2	0	–	–	–	–
Morgan et al	7	1	0	0	1	1	2	2	0	–	–	–	–
Sirkin et al	9	1	2	1	1	1	2	1	0	–	–	–	–
Zelle et al	11	2	2	1	2	1	2	1	0	–	–	–	–
Guan et al	9	1	2	1	1	1	2	1	0	–	–	–	–
Chen et al	12	2	2	1	2	1	2	2	0	–	–	–	–
Wang et al	9	1	2	0	2	1	2	1	0	–	–	–	–
Leonetti et al	12	2	2	1	2	1	2	2	0	–	–	–	–
Beckwitt et al.^a^	19	2	2	1	2	1	2	2	0	2	2	1	2

^a^Comparative study

## Discussion

Tibial pilon fractures resulting from high-energy mechanisms typically involve substantial comminution and soft tissue injury. Over the years, several treatment methods have been used for the management of these fractures [30–32]. Although there is no standardized method of fixation [33], most surgeons favor a staged approach to allow resolution of soft tissue swelling, which typically involves temporary ankle-bridging external fixation with or without fibular internal fixation followed by definitive ORIF [8, 10–15]. The current study’s aim was to determine, on the basis of a review of the current literature, whether primary ankle arthrodesis is a reasonable alternative treatment choice to staged ORIF for AO43-C3 fractures. Although our qualitative synthesis demonstrated no differences in any of the observed outcomes other than a tendency towards decreased short-term complications (wound complications, infection, or hardware pain) with arthrodesis, there was insufficient evidence to allow for any definitive conclusion. The authors found that there are relatively few quality data reported in the literature regarding the management of AO 43-C3 fractures. None of the studies reviewed had complete data, there was a lack of homogeneity in outcomes reporting, and most studies did not adequately distinguish among fracture types or report complete data on complications. Additionally, data on primary ankle arthrodesis are limited compared with staged ORIF and with all of these articles reporting only relatively small numbers of cases.

### PICO question 1: short-term complications

Using primary arthrodesis for severely comminuted pilon fractures is not a novel concept. Beckwitt et al. examined the differences in patient outcomes between primary arthrodesis and primary ORIF in AO-43C3 pilon fractures and found that patients treated with the arthrodesis approach had a lower rate of nonunion [23]. While this study was notable in that the average follow-up was 73.7 months, like the other studies included in this review, it did not have a treatment arm involving staged treatment (primary ORIF was the comparator group). Zelle et al. [22] assessed 20 patients who underwent blade plate ankle fusion of comminuted tibial plafond fractures, and with a two-year follow-up reported no wound complications and only one incidence of nonunion. Bozic et al. [8] treated 15 severely comminuted AO 43-C3 fractures with primary tibiotalar arthrodesis using a fixed-angle blade plate, achieving ankle fusion at an average of 15 weeks (range, 10–21). No patient required secondary procedures to obtain union. Similar results had previously been reported by Morgan et al. [21] in a series of 6 patients, in which primary ankle fusion was obtained after a mean of 26 weeks (range, 20–34), without any reported incidences of malunion or nonunion. The rates of malunion and nonunion are crucial aspects of this discussion, as they are among the most consequential complications of complex tibial pilon fractures. The individual rates from these studies as well as our qualitative synthesis suggest promising results for arthrodesis so far in this regard with a 2.9% rate compared to 9.9% in the staged ORIF group. However, given the limited numbers in the available literature (only two reported nonunions among patients treated with arthrodesis from the studies included in this review), further investigation of the difference in rates of nonunion and malunion is warranted, particularly with regards to infectious versus noninfectious causes.

In a retrospective study of 63 patients, Beaman and Gellman [20] reported on the outcomes of 13 patients treated over 2 years, whose highly comminuted tibial pilon fractures were treated with primary arthrodesis. The authors, who chose fusion based on clinical experience, theorized that primary ankle arthrodesis would expedite the patients’ recovery and return to regular activity and improve clinical outcomes without the need for multiple procedures and long recovery times, as required with a staged approach. In their series, they were able to support their assumption with a high healing rate and good overall American Orthopaedic Foot and Ankle Society (AOFAS) functional score of 83 [34]. These findings are compatible with the functional score seen after ankle arthrodesis for osteoarthritis. Similar results were published by Zelle et al. [22], who retrospectively reviewed 20 patients, all with AO 43-C3 fractures treated over a period of 17 years with primary arthrodesis. Their results were comparable to those of other primary arthrodesis studies, with very low soft tissue complication and nonunion rates and acceptably good outcomes, with all patients able to ambulate without assistive devices. These latest findings complement the outcome published by Hendrickx et al. [35] in 2011, who studied 66 ankle arthrodesis cases performed for various indications at a mean follow-up of 9 years. The authors found that 91% of their patients were satisfied with their achieved ankle condition, with an AOFAS score of 67 ± 12 and an improved mental health perception according to the SF-36 score [35]. On the basis of their findings, they suggested that ankle arthrodesis improves the quality of life for patients with end stage ankle osteoarthritis.

### PICO question 2: long-term complications

Poor outcomes of pilon fractures is associated with the extent of articular surface involvement and cartilage damage and quality of the anatomical reduction of the fracture fragments [3, 8, 33, 36–38]. This point has been highlighted by Anderson et al. [36] and Marsh et al. [38],who emphasized the high incidence (up to 50%) of PTOA secondary to fractures of the distal tibial articular surface. Both studies reported a strict correlation (*P* < .01) between the development of PTOA and the high-energy injury and fracture pattern, with a 20-fold higher risk of developing osteoarthritis within 2 years after high-energy pilon fractures. Similarly, Horisberger et al. [37] addressed the link between the development of PTOA and ankle fracture pattern with significantly shorter osteoarthritis latency time (*P* < .01) in highly comminuted pilon fractures. Such articles emphasize the importance of evaluating the degree of articular surface damage because it can indicate a probable poor outcome.

Unfortunately, much of the literature reviewed in this study lacks long-term data needed to assess for the development of these outcomes. Among cases involving a staged treatment protocol, the average follow-up period was 26 months (range, 9–36). This number was significantly greater in the primary arthrodesis cohort, with an average follow-up time of 60 months (range, 24–86). Follow-up length is especially important when considering outcomes like PTOA, secondary arthrodesis, and amputation because these developments are typically observed with medium- or long-term follow-up. Most of the staged approach studies reviewed focused on short- or medium-term outcomes and thus did not adequately address long-term outcomes, especially arthrosis development. On the basis of the review of staged procedure articles, with reported PTOA rates of 20 to 50%, the authors estimate the rate of secondary arthrodesis in patients with comminuted pilon fractures to be higher than the data reported in more recent series (8 to 20%) [3, 15, 26–29] as most of these studies had inadequate follow-up, an issue commonly seen in trauma populations [39]. No PTOA is expected in the primary arthrodesis group, as this complication is avoided with the joint fusion procedure. Thus, on the basis of this review, no conclusions can be made regarding the comparison of PTOA long-term outcomes between arthrodesis and staged ORIF.

### Limitations

The conclusions of this study are restricted by certain limitations, however. The arthrodesis group had a substantially higher proportion of patients with C3-type fractures in comparison to the staged ORIF group, which may represent a possible selection bias from the included studies and must be considered in any interpretation of the pooled results. Additionally, orthopaedic literature can be biased toward reporting positive outcomes [12, 40–44]. To educate their peers, surgeons tend to report methods that have led to good results, whereas most failures are not reported [40]. As a result, a cursory review of the literature may suggest that these complicated fractures are characterized by good outcomes when in reality the outcomes are not as encouraging as described in previous studies [44–46]. As discussed above, the lack of long-term data also contributes to the limitations of this study. Finally, outcomes reporting was not homogeneous between the literature included in this review, further limiting the ability to consolidate data from multiple studies. Despite these limitations, the strengths of this study include the larger sample size permitted by the consolidation of multiple studies. Most studies in the literature are small studies or based at single institutions, leading a limited sample size. A pooled analysis is often the only way to generate larger sample sizes for populations with injuries as rare as AO 43-C3 pilon fractures.

### Recommendations

Currently, staged ORIF is the recommended treatment for severely comminuted fractures of the tibial pilon, and although primary arthrodesis appears to be associated with lower morbidity and an acceptable success rate, the authors cannot make any definitive statement about the outcomes at present. The literature regarding outcomes of AO 43-C3 pilon fractures remains limited and lacks reports of long-term evaluation and PTOA, which can be difficult to evaluate with poor follow-up rates in trauma patients. Randomized clinical trials using universal and standardized reporting methods are needed to properly define the long-term outcomes and direct the care for these patients. On the basis of the available literature, similar studies should be performed for primary arthrodesis.

## Data Availability

Please contact the corresponding author for data requests.

## Abbreviations

PICO: population, intervention/exposure, comparison, outcomes
AO/OTA: Arbeitsgemeinschaft für Osteosynthesefragen/Orthopaedic Trauma Association
ORIF: open reduction and internal fixation
PTOA: post-traumatic osteoarthritis
PRISMA: Preferred Reporting Items for Systematic Reviews and Meta-Analyses
RCTs: randomized controlled trials
MINORS: methodological index for non-randomized studies
AOFAS: American Orthopaedic Foot and Ankle Society
